# Correction to “Exploring the Association Between Systemic Lupus Erythematosus and High‐Density Lipoproteins: A Systematic Review and Meta‐Analysis”

**DOI:** 10.1002/acr2.90138

**Published:** 2026-09-01

[ Pérez‐Ocampo, J. , Taborda, N.A. , Yassin, L.M. , Higuita‐Gutiérrez, L.F. and Hernandez, J.C . (2024), Exploring the Association Between Systemic Lupus Erythematosus and High‐Density Lipoproteins: A Systematic Review and Meta‐Analysis. ACR Open Rheumatology, 6: 648‐661. 10.1002/acr2.11700]39030864 PMC11471950

In the byline for Exploring the Association Between Systemic Lupus Erythematosus and High‐Density Lipoproteins: A Systematic Review and Meta‐Analysis, the affiliations for author Hernandez, JC were incorrect. The correct affiliation is Infettare, Facultad de Medicina, Universidad Cooperativa de Colombia, Medellin, Colombia.

We apologize for this error.

